# Biofilm growth mode promotes maximum carrying capacity and community stability during product inhibition syntrophy

**DOI:** 10.3389/fmicb.2014.00693

**Published:** 2014-12-15

**Authors:** Kristen A. Brileya, Laura B. Camilleri, Grant M. Zane, Judy D. Wall, Matthew W. Fields

**Affiliations:** ^1^Department of Microbiology and Immunology, Montana State UniversityBozeman, MT, USA; ^2^Center for Biofilm Engineering, Montana State UniversityBozeman, MT, USA; ^3^Division of Biochemistry, University of MissouriColumbia, MO, USA; ^4^Thermal Biology Institute, Montana State UniversityBozeman, MT, USA; ^5^Ecosystems and Networks Integrated with Genes and Molecular AssembliesBerkeley, CA, USA; ^6^National Center for Genome ResourcesSanta Fe, NM, USA

**Keywords:** anaerobic, carrying capacity, hydrogen transfer, population intermixing, sulfate-reducing bacteria

## Abstract

Sulfate-reducing bacteria (SRB) can interact syntrophically with other community members in the absence of sulfate, and interactions with hydrogen-consuming methanogens are beneficial when these archaea consume potentially inhibitory H_2_ produced by the SRB. A dual continuous culture approach was used to characterize population structure within a syntrophic biofilm formed by the SRB *Desulfovibrio vulgaris* Hildenborough and the methanogenic archaeum *Methanococcus maripaludis*. Under the tested conditions, monocultures of *D. vulgaris* formed thin, stable biofilms, but monoculture *M. maripaludis* did not. Microscopy of intact syntrophic biofilm confirmed that *D. vulgaris* formed a scaffold for the biofilm, while intermediate and steady-state images revealed that *M. maripaludis* joined the biofilm later, likely in response to H_2_ produced by the SRB. Close interactions in structured biofilm allowed efficient transfer of H_2_ to *M. maripaludis,* and H_2_ was only detected in cocultures with a mutant SRB that was deficient in biofilm formation (Δ*pilA*). *M. maripaludis* produced more carbohydrate (uronic acid, hexose, and pentose) as a monoculture compared to total coculture biofilm, and this suggested an altered carbon flux during syntrophy. The syntrophic biofilm was structured into ridges (∼300 × 50 μm) and models predicted lactate limitation at ∼50 μm biofilm depth. The biofilm had structure that likely facilitated mass transfer of H_2_ and lactate, yet maximized biomass with a more even population composition (number of each organism) when compared to the bulk-phase community. Total biomass protein was equivalent in lactate-limited and lactate-excess conditions when a biofilm was present, but in the absence of biofilm, total biomass protein was significantly reduced. The results suggest that multispecies biofilms create an environment conducive to resource sharing, resulting in increased biomass retention, or carrying capacity, for cooperative populations.

## INTRODUCTION

Symbiosis (“living together”) and specifically mutualism, whereby both parties incur a benefit from living together, is widespread throughout the biosphere with well-studied examples in and across all three domains of life (Yeoh et al., 1968; Boucher, 1988; Kato et al., 2011; Moissl-Eichinger and Huber, 2011; Plugge et al., 2011; Gokhale and Traulsen, 2012; Sieber et al., 2012). In communities of bacteria and archaea, mutualism is typically referred to as syntrophy (“eating together”) where by-products of one metabolism serve as substrates for another metabolism (Sieber et al., 2012). The syntrophy between sulfate-reducing bacteria (SRB) and methanogenic archaea is of interest because these guilds both play crucial roles in many different anaerobic environments. SRB link the carbon, sulfur, and nitrogen biogeochemical cycles via carbon-oxidation and sulfate reduction (Purdy et al., 2002; Canfield et al., 2010) and also contribute to redox gradients of microbial ecosystems via the production of sulfide compounds (Moreau et al., 2010; Xie et al., 2013). Methanogenic archaea are responsible for the three largest sources of methane flux to the atmosphere (wetlands, ruminants, and rice cultivation) and form the basis of most anaerobic environments (natural and man-made) that convert CO_2_, H_2_, and/or acetate/methyl-groups to methane (Thauer et al., 2008; Neef et al., 2010; van Groenigen et al., 2012; Schlesinger and Bernhardt, 2013).

The nature of SRB-methanogen interactions is complex and fluctuates based on substrate flux and availability (Leloup et al., 2009; Stams and Plugge, 2009; Plugge et al., 2011). In the presence of sulfate, methanogens are typically outcompeted by SRB using H_2_, formate, and acetate as electron donors for sulfate reduction (Plugge et al., 2011). SRB can alternatively form mutualistic partnerships with hydrogenotrophic methanogens in the absence of sulfate by proton reduction to form H_2_ gas. The reaction is kept favorable when hydrogenotrophic methanogens consume H_2_, keeping the partial pressure low and thereby eliminating the inhibitory effect of this end-product on the SRB (Figure S1A; McInerney et al., 2009; Stams and Plugge, 2009). Inhabitants of anaerobic ecosystems are assumed to function at the thermodynamic limit for energy generation and biomass production given system constraints (Bryant et al., 1977; Thauer et al., 2008; McInerney et al., 2009; Kato and Watanabe, 2010). When one metabolism is obligately coupled to another through interspecies H_2_, formate, or electron transfer, organisms must persist by sharing the overall free energy of the reaction (Kato and Watanabe, 2010). Therefore, syntrophic physiology plays an important role in microbial communities dominated by fluctuations in nutrient availability and stress, where community interactions are thought to provide stability (Hansen et al., 2007).

It is well-accepted that microorganisms can exist attached to surfaces and each other, often surrounded by extracellular polymeric substances (EPS; Hall-Stoodley et al., 2004; Gross et al., 2007; Stewart and Franklin, 2008). Biofilms have been described from environments where there are liquid–solid or liquid–gas interfaces that include terrestrial and deep-sea hydrothermal features, riparian zones, ship hulls, metal pipes, saturated soils, and the human body (Hall-Stoodley et al., 2004), but much of the work to identify the driving force and genetic control over biofilm formation has been done with pure culture studies (e.g., Gross et al., 2007; Stewart and Franklin, 2008; Perez-Osorio et al., 2010; Clark et al., 2012).

The structure and function of multispecies biofilms can be more complex than monocultures, and biofilm structure from several environments has been characterized with confocal scanning laser microscopy (CSLM) using fluorescence *in situ* hybridization (FISH) and immunofluorescence (Møller et al., 1998; Hall-Stoodley et al., 2006; Al-Ahmad et al., 2009; Stams and Plugge, 2009; Jakubovics, 2010; Zijnge et al., 2010). We have recently shown that the structure of a mixed biofilm community is dependent upon the nature of the interactions (i.e., cooperative or competitive) and that the degree of intermixing of two-member communities is greater during cooperation versus competition (Momeni et al., 2013). The dependence of biofilm structure on function has also been demonstrated in a dual culture system where commensal biofilm cells interacted closely in mixed micro-colonies, while non-commensals formed separate non-interacting biofilm micro-colonies (Nielsen et al., 2000). Despite the ubiquity of biofilms and importance of anaerobes, little work has been done to understand how biofilm structure affects function in anaerobic microbial communities (Raskin et al., 1996; Nielsen et al., 2000; Brenner and Arnold, 2011; Bernstein et al., 2012).

While interactions between SRB and methanogens have been studied, very little has been done to characterize the emergent properties of interactive populations in anaerobic biofilms. The purpose of this work was to characterize the relationship between biofilm structure and function in biofilm formed by a SRB and a hydrogenotrophic methanogen cultured syntrophically under nutrient limitation and nutrient excess conditions (i.e., carbon source and electron donor/acceptor). We hypothesized that a biofilm would be functionally more efficient in terms of product formation (i.e., CH_4_) compared to populations in the bulk aqueous phase. A system was developed for anaerobic continuous culture where biofilm and planktonic growth phases could be monitored to determine the difference in biomass yield per mass flux of lactate and methane under varying conditions. To compare the biomass yield of biofilm to the biomass yield of planktonic cocultures, we removed the biofilm from a series of reactors, and in another experiment, used a biofilm deficient mutant coculture.

## MATERIALS AND METHODS

### CULTURE CONDITIONS

*Desulfovibrio vulgaris* ATCC 29579 and *Methanococcus maripaludis* S2 (DSM 14266) were continuously cultured in modified 1L CDC reactors (BioSurface Technologies Corp., Bozeman, MT, USA) for anaerobic biofilm growth (Figure S1B). Biofilm coupon holders were modified to hold glass microscope slides cut to 7.6 cm × 1.8 cm as previously described (Clark et al., 2012). Both monocultures and cocultures were grown in coculture medium (CCM), a bicarbonate buffered, basal salts medium without choline chloride (Walker et al., 2009). Monoculture *D. vulgaris* medium was supplemented with 25 mM sodium sulfate, or grown in standard lactate-sulfate medium (LS4D) with 30 mM lactate and 25 mM sodium sulfate as previously described Clark et al. (2006). Headspace (290 mL) was purged at 20 mL/min with anoxic 80% N_2_:20% CO_2_ (v/v) for coculture and monoculture *D. vulgaris* or 80% H_2_:20% CO_2_ for monoculture *M. maripaludis* through a 0–20 SCCM mass controller (Alicat Scientific, Tucson, AZ, USA). Reactors were maintained at 30°C with stirring at 80 rpm. The reactor aqueous phase (375 mL) was inoculated with 20 mL of mid-exponential phase planktonic cultures grown from glycerol freezer (–80°C) stocks in 40 mL of CCM in 125 mL serum bottles. Fresh CCM in a 20 L glass carboy was continuously sparged with sterile anoxic 80% N_2_:20% CO_2_ and supplied at a dilution rate of 0.017 h^-1^ starting after 48 h of batch growth by a Masterflex L/S pump (Cole-Parmer Instruments Co., Vernon Hills, IL, USA). Batch monoculture *M. maripaludis* was grown in Balch tubes in 5 mL of CCM with 30 mM acetate in lieu of lactate, prepared under 80% N_2_:20% CO_2_, and then pressurized after autoclaving to 200 kPa with 80% H_2_:20% CO_2_.

### GAS CHROMATOGRAPHY

Gas measurements were made by automated injections (250 ms) of reactor headspace via a 16-port stream selector (Vici-Valco Instruments Co. Inc., Houston, TX, USA) to a 490microGC (Agilent Technologies Inc., Santa Clara, CA, USA) equipped with dual channels and dual thermal conductivity detectors. Molsieve5A and PoraplotQ (both 10 m) columns were run with Helium carrier gas at 145 kPa and 80°C with injectors at 110°C and heated sample line at 40°C. The CDC reactor lids were fitted with stainless steel fittings (Swagelok, Idaho Falls, ID, USA) to accommodate 1/16” PEEK tubing to the stream selector. Scotty calibration gasses were used as standards (Air Liquide America Specialty Gases, Plumsteadville, PA, USA).

### FLUORESCENCE *IN SITU* HYBRIDIZATION

Fluorescence *in situ* hybridization was used on scraped biofilms to determine relative biovolume of each cell type. Whole biofilm on the glass coupon was fixed in 4% paraformaldehyde in a 50 mL conical tube for 3 h at 4°C, then scraped into a well on a Teflon coated slide (Paul Marienfeld GmbH & Co. KG, Lauda-Königshofen, Germany). Dried biofilm was dehydrated and hybridized in buffer solution containing 180 μL 5 M NaCl, 20 μL 1 M Tris HCl, 449 μL double deionized (dd) H_2_O, 1 μL 10% SDS and 350 μL deionized formamide (final concentration 35%) with 3 ng each of probes EUB338 (GCT GCC TCC CGT AGG AGT) double labeled with Cy3 and ARCH915 (GTG CTC CCC CGC CAA TTC CT) double labeled with Cy5 for 4 h at 46°C in a humid chamber (Stoecker et al., 2010). Samples were washed in prewarmed washing buffer containing 700 μL 5 M NaCl, 1 mL 1 M TrisHCl, 500 μL 0.5 M EDTA and raised to 50 mL with ddH_2_O, at 47°C for 10 min, then dipped in ice cold ddH_2_O and quickly dried with compressed air. Samples were mounted with Citifluor AF1 antifadent (Citifluor Ltd., Leicester, UK) for CLSM. 3D-FISH (Daims et al., 2006) was used to determine colocalization patterns on intact, unscraped biofilm. For 3D-FISH, whole fixed biofilm on the glass coupons was embedded in polyacrylamide prior to dehydration (Daims et al., 2006; Brileya et al., 2014).

### CONFOCAL LASER SCANNING MICROSCOPY

Fluorescently labeled biofilm was imaged using a Leica TCS SP5 II inverted confocal laser scanning microscope with 488, 561, and 633 nm lasers and appropriate filter sets for Cy3 and Cy5. Polyacrylamide-embedded whole biofilm for 3D-FISH and fluorescently stained hydrated biofilm were imaged on a Leica TCS SP5 II upright confocal laser scanning microscope using a 63x 0.9 NA long working distance (2.2 mm) water dipping objective (Leica Microsystems, Exton, PA, USA).

### 5-CYANO-2,3-DITOLYL TETRAZOLIUM CHLORIDE (CTC) STAINING

Biofilm metabolic potential was assessed using the redox stain 5-cyano-2,3-ditolyl tetrazolium chloride (CTC). Whole hydrated biofilm coupons were removed in an anaerobic chamber and incubated in freshly prepared anoxic 0.05% CTC solution for 2 h as in Stewart et al. (1994). The reaction was stopped with 5% formaldehyde and rinsed with ddH_2_O. Hydrated biofilm was stained with 1 μg/mL DAPI for 20 min in the dark and rinsed with ddH_2_O before CLSM.

### CELL COUNTS AND BIOFILM RELATIVE ABUNDANCE

One milliliter of planktonic phase was fixed in formaldehyde (final concentration 2%) overnight then diluted as necessary and stained for 20 min in the dark with an equal volume of filtered 0.3g/L Acridine Orange. Stained samples were collected through a filter chimney on a black polycarbonate track-etched isopore filter (EMD Millipore Corp., Billerica, MA, USA) and imaged on a Nikon Eclipse E800 microscope with a mercury bulb for fluorescence. At least ten random fields of view were analyzed and cells were counted via integrated morphometry analysis in MetaMorph version 7.6 (Molecular Devices, Sunnyvale, CA, USA).

Biofilm relative abundance (biovolume) was determined using thirty CLSM images per sample, captured from random locations in x, y, and z planes. MetaMorph was used to measure the thresholded area of the two channels in each image.

### PROTEIN NORMALIZATION

One *M. maripaludis* cell and one *D. vulgaris* cell are not the same shape or volume, so average biomass (protein) per cell was determined using monocultures. Biological duplicates of each monoculture were grown to late exponential phase in 125 mL serum bottles. One portion was filtered and dried to determine dry weight per cell. The Lowry protein assay was done in triplicate on each culture to determine protein biomass weight per volume. Additionally cells were fixed and stained for counting as described above. Twenty fields of view were analyzed for each culture to determine cell number per volume and area per cell using MetaMorph version 7.6 software (Figure S2). Protein per cell area was observed to be equivalent in both cell types on average, so no correction factor was applied when determining the fraction of biofilm biovolume contributed by *M. maripaludis* and *D. vulgaris*. Total protein per cell was found to be skewed toward one cell of *D. vulgaris* containing more protein than one cell of *M. maripaludis* (Figure S2). Therefore when a total planktonic protein measurement was related to cell counts of each population, a correction factor was also applied where 40% of one protein unit was attributed to *M. maripaludis* and 60% to *D. vulgaris*.

### 1-D BIOFILM ACCUMULATION MODEL

Diffusion in the biofilm was modeled using a biofilm accumulation model (BAM; Wanner and Gujer, 1986) to predict effects of biofilm thickness, inlet substrate concentration, and volumetric flow rate on methane production and cell ratios. Input parameters are listed in Table S1. Rate coefficients for substrates were K_s_ = 1 while stoichiometric coefficients were 1/yield. Yields were calculated based on Gibbs Free Energies for the associated half reactions normalized to one electron. Aqueous diffusion coefficients (D_aq_) at 25°C for substrates (Stewart, 2003) were corrected to 30°C using D_30_/D_25_ = 1.135. D_aq_ of lactate was calculated as in Wilke and Chang (1955):

$$\frac{D_{L}\,\mu }{T_{a\,b\,s}}=\frac{-7.4\times 10^{-8}\,{\left(X\,M\right)}^{0.5}}{V_{b}^{0.6}}$$

Biofilm accumulation model allows for input of a ratio of the effective diffusion coefficient to the aqueous diffusion coefficient (D_e_/D_aq_) which is then applied to all solutes to account for the decreased diffusion observed in the biofilm matrix compared to water.

### ELECTRON MICROSCOPY

**FIGURE 1:**
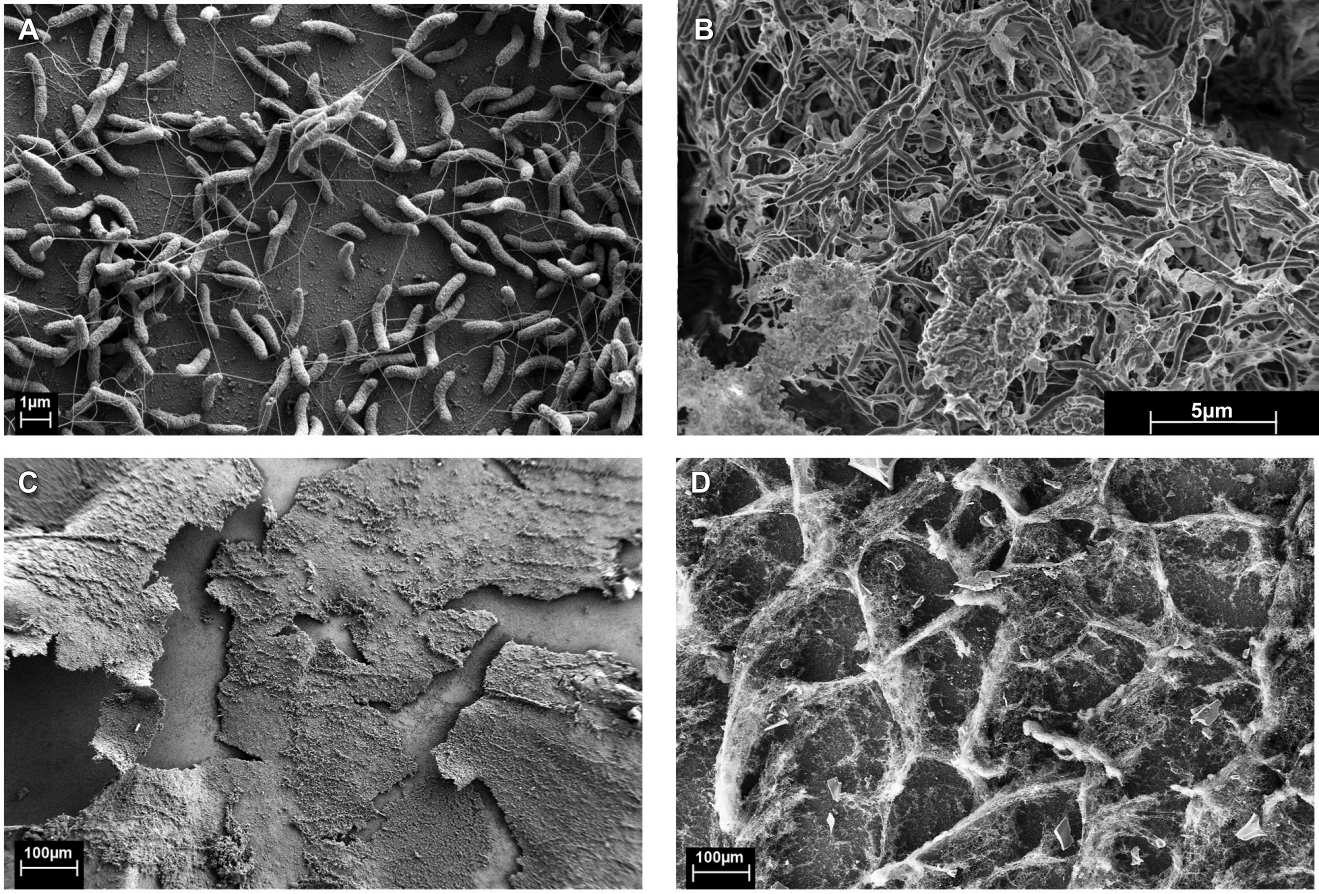
Monocultures of **(A,C)** *Desulfovibrio vulgaris* biofilm from continuous culture (5,420X and 101X, respectively). **(B,D)** Coculture biofilm of *D. vulgaris* and *M. maripaludis* (10,000X and 100X, respectively).

Micrographs in **Figures 1A,C,D and 3** were collected on a Zeiss Supra55VP FE-SEM. Biofilm was fixed in a solution of 2% paraformaldehyde, 2.5% glutaraldehyde and 0.05 M Na-cacodylate overnight at room temperature. Coupons were rinsed and stepwise dehydrated in ethanol before being cut and critical point dried on a Samdri-795 (Tousimis Research Corporation, Rockville, MD, USA). Glass pieces with dry biofilm were mounted on SEM stubs with double-sided carbon tape and silver, then sputter coated with Iridium for 35 s at 35 mA.

**Figure 1B** was unfixed biofilm scraped directly onto double-sided carbon tape, frozen while hydrated in liquid N_2_, splutter-coated with Platinum for 2 min, and imaged using a dual beam focused ion beam (FIB)-FE-SEM (Helios NanoLab, FEI Company, Hilsboro, OR, USA) equipped with a cryostage.

### SAMPLE COLLECTION AND ANALYSIS

Reactor outflow was collected on ice and measured daily to monitor flow rate. Samples of the planktonic phase were collected at the outflow for optical density at 600 nm (OD), high performance liquid chromatography (HPLC), protein measurements, and direct cell counts. Filtered samples were analyzed in triplicate with a fucose internal standard, for lactate, acetate, and formate concentrations via HPLC (Agilent 1200 series) equipped with a BioRad Aminex HPX-87H column. Lactate and formate concentration were measured with a VWD detector while acetate concentration was measured with an RID detector. Planktonic cultures were centrifuged at 6,000 *g* for 10 min and the whole cell pellet was analyzed for protein, hexose, pentose, and uronic acid composition. Biofilm samples for these analyses were collected by aseptically replacing a biofilm coupon with a sterile butyl stopper, and scraping the biofilm into sterile water with a spatula. Whole biofilm was analyzed for protein and carbohydrates. Protein concentrations were determined with the Lowry et al. (1951) assay using bovine serum albumin as the standard. Hexose sugars were measured by the colorimetric cysteine–sulfuric acid method with glucose as the standard. Pentose sugars were measured with a colorimetric orcinol-FeCl_3_ assay with xylose as the standard. A colorimetric carbazole assay was used to measure uronic acid concentration with D-galacturonic acid as the standard (Chaplin and Kennedy, 1994).

## RESULTS

### BIOFILM STRUCTURE AND COMPOSITION

Monocultures of *D. vulgaris* formed biofilm on silica slides under continuous culture conditions when sulfate was provided as an electron acceptor (**Figures 1A,C**). Monoculture *M. maripaludis* did not form a biofilm on silica slides when grown in continuous culture supplemented with H_2_, as observed with protein assay, light microscopy, and scanning electron microscopy. Material was observed on the glass slides but was confirmed to be salts via energy dispersive X-ray spectroscopy and not protein or carbohydrate (data not shown).

When *D. vulgaris* and *M. maripaludis* were cocultivated with lactate (without sulfate and H_2_), methane was produced and biofilm was formed. The coculture biofilm had an altered appearance and structure compared to monoculture *D. vulgaris* biofilm (compare **Figures 1A to 1B and 1C to 1D**) and *M. maripaludis* cells were observed in both the biofilm and planktonic phases. These results indicate that the methanogen was dependent upon *D. vulgaris* under the tested conditions to grow in a biofilm state.

**FIGURE 2:**
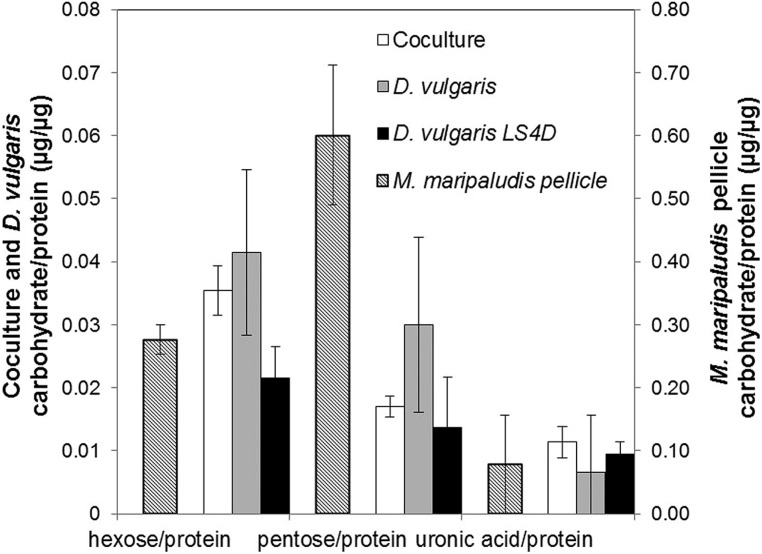
**Biofilm carbohydrate composition normalized to protein biomass for hexose, pentose, and uronic acid in continuous culture biofilm** (coculture and *D. vulgaris* monoculture, primary axis) and batch *M. maripaludis* pellicle (secondary axis). Error bars represent 95% confidence interval. Coculture in CCM *n* = 6, *D. vulgaris* in CCM *n* = 4, *D. vulgaris* in LS4D *n* = 8, *M. maripaludis* batch pellicle in CCM *n* = 3.

The protein and carbohydrate levels were compared for different growth conditions (cell-associated carbohydrate levels were normalized to protein biomass). As previously reported, *D. vulgaris* does not produce an extensive carbohydrate-rich biofilm on glass slides (Clark et al., 2007), but in CCM, *D. vulgaris* produced slightly increased levels of hexose and pentose equivalents compared to growth in LS4D medium (**Figure 2**). The uronic acid levels were similar for *D. vulgaris* when grown in LS4D or CCM (**Figure 2**) under the tested conditions. In the coculture biofilms, the uronic acid levels were similar while hexose and pentose levels were slightly decreased compared to *D. vulgaris* monocultures in CCM (**Figure 2**). The reported values were lower than previous reports for other monoculture and multispecies biofilm EPS that can constitute as much as 90% of the dry mass of a culture (Flemming and Wingender, 2010; Poli et al., 2010). Lack of extracellular material might present less mass transfer resistance to H_2_ diffusion and would therefore be beneficial to both organisms. As noted above, *M. maripaludis* did not form monoculture biofilm under the tested conditions; however, *M. maripaludis* did form a pellicle when grown in static tubes as a monoculture. The *M. maripaludis* pellicle had approximately 10-fold more uronic acid, 7-fold more hexose, and 30-fold more pentose compared to coculture biofilm (**Figure 2**). These results suggest that *M. maripaludis* had altered carbon flow that resulted in less carbon allocation to EPS.

**FIGURE 3:**
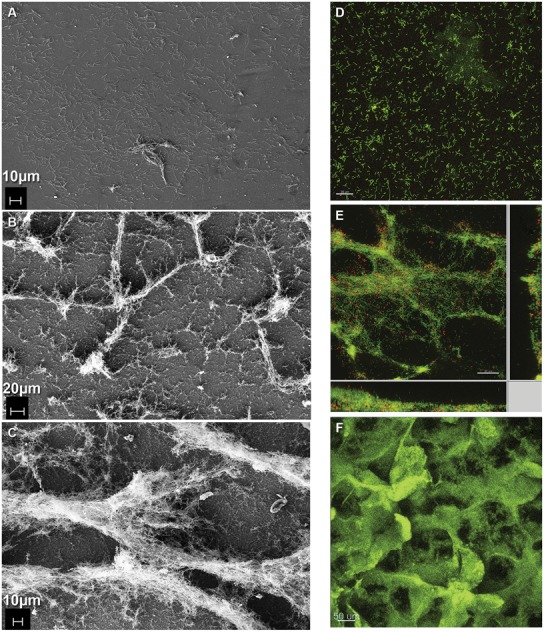
**Formation of coculture biofilm over time. (A–C)** Electron micrographs of fixed coculture at **(A)** early (386X, 0 h; **B**) intermediate (243X, 48 h; **C**) steady-state (336X, 240 h) time points. **(D–F)** Fluorescence micrographs of coculture biofilm **(D,E)** embedded in polyacrylamide and hybridized with domain-specific oligonucleotide probes labeled green for *D. vulgaris* and red for *M. maripaludis* at **(D)** early (0 h) and **(E)** intermediate (48 h) time points. **(F)** Intact hydrated biofilm unfixed and stained with Acridine Orange at steady-state (240 h).

### COCULTURE BIOFILM DEVELOPMENT AND STRUCTURE

Coculture biofilm was initiated by *D. vulgaris,* which formed a monolayer during the initial 48 h of batch mode in the continuous culture system (**Figures 3A,D**). At this early time point (0 h, initiation of flow) *D. vulgaris* out-numbered *M. maripaludis* in the biofilm 32:1, while the planktonic phase ratios of *D. vulgaris* to *M. maripaludis* were 2.7:1 (**Table 1**). After 48 h of continuous culture the biofilm grew in ridges, both normal and parallel to flow caused by liquid agitation (**Figures 3B,E**). Cell ratios in the biofilm decreased rapidly to 3.5:1 after 48 h as *M. maripaludis* cells were incorporated into the biofilm and grew, while planktonic ratios increased slightly to an average of 3.2:1. After 240 h, steady-state ratios of cells in the biofilm remained approximately 2.2:1 with similar planktonic ratios of 1.6:1 (**Table 1**). This is in contrast to the published 4:1 ratios observed in planktonic-only reactors for the same syntrophic pair under lactate-excess conditions and our own observation of 6.3:1 in planktonic phase-only reactors (**Table 1**; Stolyar et al., 2007; Walker et al., 2009, 2012). These results indicate that four times more *D. vulgaris* are typically needed to oxidize enough lactate to generate sufficient H_2_ for *M. maripaludis* in the bulk aqueous phase; however, in the presence of biofilm, a more even distribution (approximately 2:1) of interacting populations was sustained in both the biofilm and planktonic phases.

**Table 1:** **Percent of *M. maripaludis* and *D. vulgaris* cells in planktonic and biofilm phases over time** [row 1 early (0 h), 2 intermediate (48 h), 3 steady-state (240 h)] in a reactor containing both growth phases, a reactor where biofilm has been removed (row 4), a reactor with an increased loading rate (row 5), and a reactor containing a coculture of Δ*pilA* mutant *D. vulgaris* with wild-type *M. maripaludis* (row 6).

Rows		% *M. maripaludis* biofilm	% *D. vulgaris* biofilm	% *M. maripaludis* planktonic	% *D. vulgaris* planktonic
1	Biofilm and planktonic early	3% ± 0.8	97% ± 0.8	26% ± 13.7	69% ± 11.1
2	Biofilm and planktonic intermediate	22% ± 1.5	78% ± 1.5	23% ± 12.1	73% ± 10.6
3	Biofilm and planktonic steady state	31% ± 1.4	69% ±1.4	36% ±10.8	59% ±2.2
4	WT planktonic only	0	0	13% ± 1.9	82% ± 1.9
5	Biofilm and planktonic increased loading rate	18% ± 1.5	82% ± 1.5	22% ± 5.9	73% ± 3.3
6	Δ*pilA* planktonic only	0	0	19% ± 1.4	81% ± 1.4

*Error is the 95% confidence interval. The number of images analyzed for each time point is early *n* = 146, intermediate *n* = 269, steady-state *n* = 2,118, biofilm removed *n* = 140, increased loading rate *n* = 174, and Δ*pilA n* = 120.*

Coculture biofilm macrostructure was observed with both fixed and unfixed, still hydrated biofilm (**Figures 1D and 3C,F**). The structured biofilm included tall ridges and spires with deep valleys, often 300–400 μm tall, but always with at least one dimension <50 μm as measured by fluorescence microscopy of intact hydrated biofilm or cryosections of frozen hydrated biofilm (**Figure 3F**). Notably, the macrostructure was not observed in *D. vulgaris* monoculture biofilms grown in LS4D medium (Clark et al., 2007) nor in CCM (**Figures 1A,C**). The critical biofilm thickness that would allow for diffusion of 30 mM lactate to the substratum was estimated to be 50 μm, as predicted by a 1D BAM (Figure S3 and Table S1). These results suggest that the macrostructure was influenced by lactate diffusion limitation. Microcolonies of *M. maripaludis* cells were spread throughout a matrix of *D. vulgaris* cells with an intermixed pattern. Cell association in the structured biofilm was observed to be random with no pattern of colocalization detectable (Figure S4). It has recently been shown that increased intermixing is a marker of cooperation (Momeni et al., 2013), so it is reasonable that this cooperative community was highly intermixed.

### BIOFILM AND PLANKTONIC COMMUNITY FUNCTION: THE BASE CASE

**FIGURE 4:**
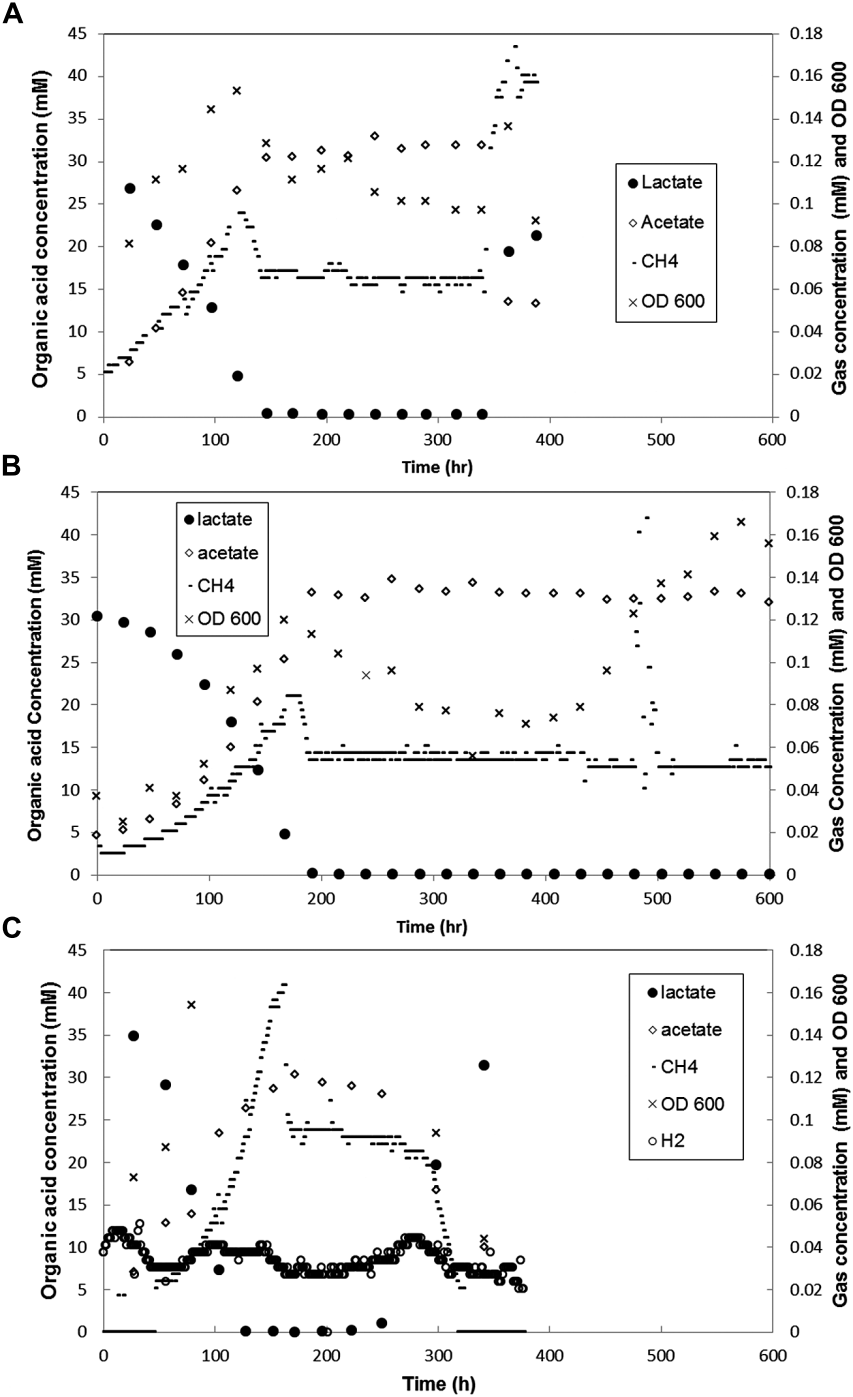
**Metabolite concentration over time in coculture biofilm reactors (A)** with both biofilm and planktonic phases in which loading rate was increased at 341 h, **(B)** that had biofilm coupons removed at 432 h **(C)** grown with Δ*pilA* mutant *D. vulgaris* and wild-type *M. maripaludis*. Each graph is representative of one of two duplicate experiments.

The base case represents the standard syntrophic system described above that contained a structured biofilm and a planktonic phase in continuous culture. Little, if any previous work has been done to characterize syntrophic interactions with interacting biofilm and planktonic phases, so a baseline understanding of function (CH_4_ and H_2_ production and lactate consumption) was necessary to determine a basal state under the tested conditions. During the first 100 h of biofilm development, methane levels increased as lactate levels declined with an equimolar increase in acetate (**Figure 4A**). The biofilm population was 78% *D. vulgaris* and 22% *M. maripaludis* (3.5:1) with similar planktonic population distribution (**Table 1**). During the next 50 h, the system approached a steady-state in which all 30 mM of lactate was consumed, and both organisms in both phases of the reactor increased rapidly in number. As lactate became limiting, the OD and methane concentration peaked for one retention time of the reactor, and then declined to a steady-state. The methane concentration stabilized but OD continually decreased while biofilm biomass increased for another 100 h after lactate was not detectable. These results indicate that biofilm cells were competitive for bulk-phase lactate, and that the biofilm growth mode contributed to more efficient, multi-species substrate utilization. This is further demonstrated in that H_2_ was not detectable in the reactor headspace at any point. Walker et al. (2009) observed a spike (50 Pa) followed by a constant low level of H_2_ (less than 10 Pa) at steady-state when a planktonic-only system was not limited for lactate. Presumably all H_2_ produced in our biofilm reactor was efficiently consumed. It should also be noted that H_2_ was not detected when the lactate loading rate was increased (discussed below).

**FIGURE 5:**
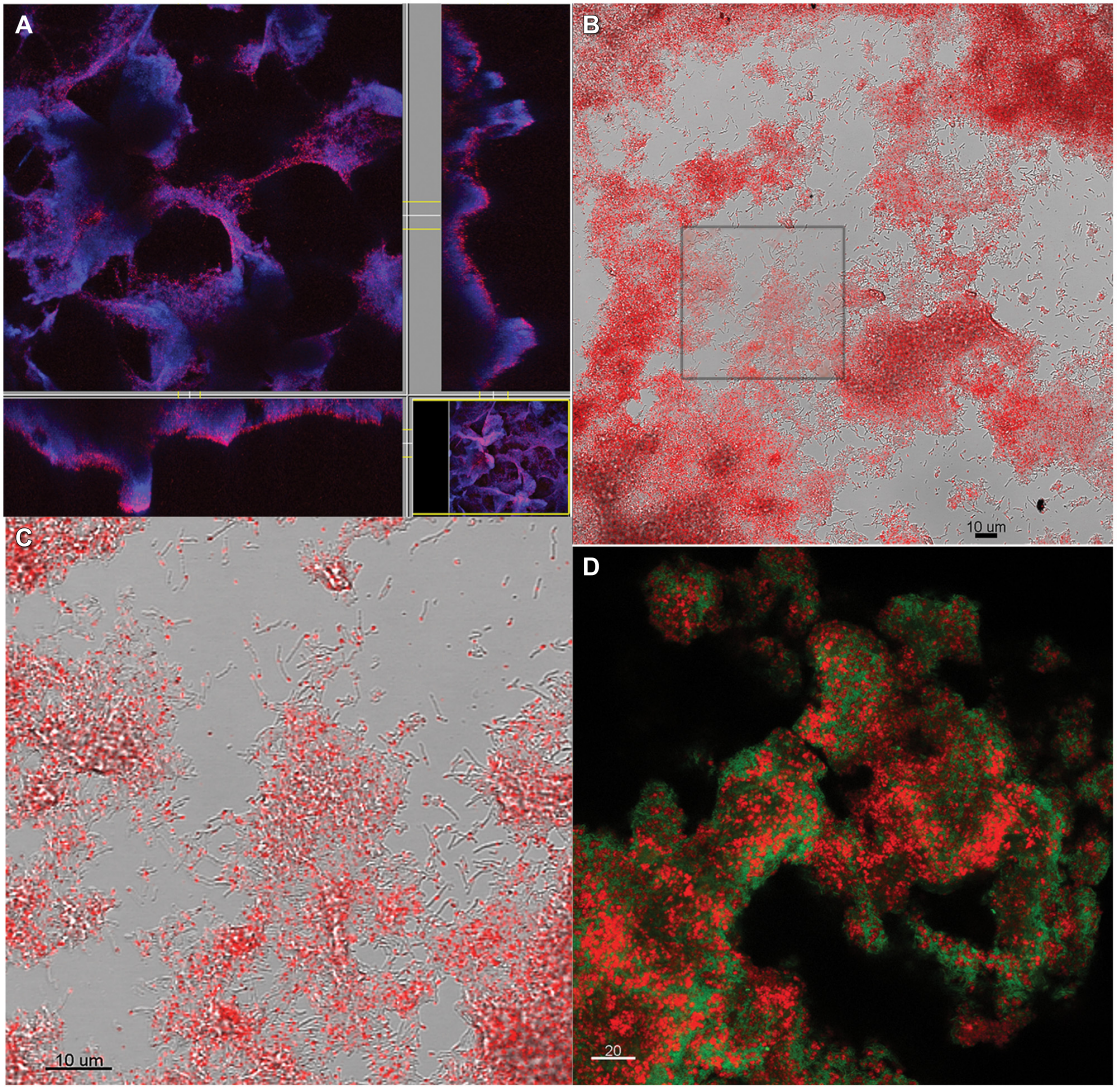
**Coculture biofilm (A)** stained with CTC while intact and hydrated and showing all biomass stained with DAPI in blue, and CTC in red, or purple where both DAPI and CTC are present **(B)** scraped from the slide after CTC staining. **(C)** Zoomed in from the inset in **(B)** showing individual grains of red fluorescent CT-formazan in each cell. **(D)** Coculture biofilm scraped from the substratum, fixed and hybridized with domain-specific probes for *D. vulgaris* (green) and *M. maripaludis* (red).

In aerobic biofilms, it has been shown that cells near the substratum can be limited in oxygen and metabolically inactive, i.e., not all the biofilm biomass is active (Xu et al., 2000). To assess the metabolic state of the syntrophic biofilm, intact, steady-state coculture biofilm was incubated with CTC (Stewart et al., 1994). The validity of this method for anaerobes has been debated with the primary concern that CTC is abiotically reduced in the presence of sulfide and cysteine (Stewart et al., 1994; Gruden et al., 2003; Halan et al., 2012). CT-formazan granules formed abiotically are poorly localized and rapidly photo-bleach, while CT-formazan of biogenic origin is an intracellular granule that is more resistant to photo-bleaching (Gruden et al., 2003). CCM contains 1 mM each of sodium sulfide and cysteine, but the biofilm was rinsed anoxically prior to staining in an anaerobic glovebag. The incubated biofilm was directly observed with CSLM, and the biofilm biomass showed respiratory potential based on formation of CT-formazan. However, portions of the intact biofilm were not visible via CSLM due to depth limitations (**Figure 5A**), therefore the biofilm was scraped for visualization (post-staining). Upon inspection of scraped biofilm, the entire biofilm biomass was stained (**Figures 5B,C**), and reduced CT-formazan granules could be observed in nearly all cells, localized inside the cell with persistent fluorescence. These results indicate that the entire steady-state biofilm biomass retained respiratory potential and was metabolically active. The same results were obtained using FISH, where all biofilm cells exhibited strong fluorescence irrespective of location, and these results corroborated the idea that all cells were active (**Figure 5D**).

### INCREASED LOADING RATE

**FIGURE 6:**
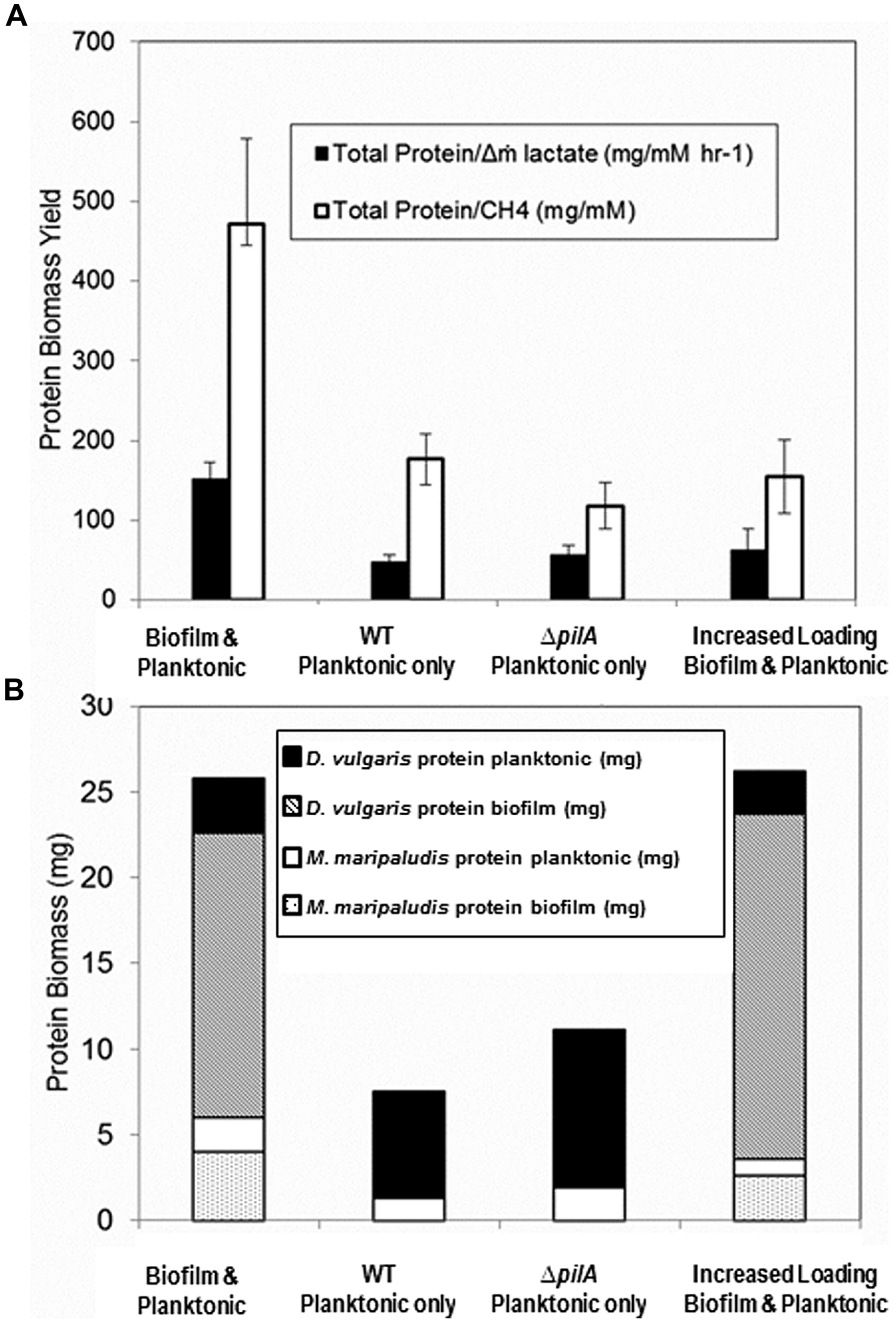
**(A) Biomass yield per lactate mass flux and per methane** for reactors containing coculture with biofilm and planktonic phases (base case; *n* = 8), planktonic phase only after biofilm was removed (*n* = 14), Δ*pilA* mutant coculture planktonic phase only (*n* = 7), and both biofilm and planktonic phases at an increased loading rate (*n* = 2). Error bars represent 95% confidence interval. **(B) Biomass distribution of each cell type in biofilm and planktonic phases for the same culture conditions based upon cell volume ratios and cell counts respectively**. Unpaired two-tailed *t*-test results indicate total protein/Δm lactate difference is significant between coculture biofilm and planktonic (base case) versus all other conditions (*p* < 0.005), but that planktonic only versus increased loading rate is not significant (*p* = 0.4). Total Protein/CH_4_ unpaired two-tailed *t*-test results were significant between biofilm and planktonic (base case) versus all other conditions [versus WT planktonic and Δ*pilA* planktonic (*p* < 0.005), biofilm and planktonic (base case) versus increased loading rate (*p* = 0.05), planktonic only versus increased loading rate (*p* = 0.4)].

To test the effect of a sudden input of nutrients on a stable community, the dilution rate was increased after 341 h to 0.109 h^-1^ (approximately 6-fold) in a biofilm reactor in steady-state. Methane levels increased within 1 h and peaked at ∼0.17 mM after 27 h (**Figure 4A**). The optical density increased slightly and then declined to just below steady-state levels, with an ∼20% decrease of *D. vulgaris* cells and ∼15% decrease in *M. maripaludis* cells in the planktonic phase based on cell counts. The system was monitored for four retention times (RT = 9.9 h) with biofilm samples removed after 48 h, and the decreased planktonic biomass is likely a result of washout. The doubling time of *D. vulgaris* in CCM supplemented with sulfate is approximately 20 h (*k* = 0.04 h^-1^) while the doubling time of *M. maripaludis* with unlimited H_2_ in CCM is 5 h (*k* = 0.14 h^-1^). Under these conditions as *D. vulgaris* was washed out of the reactor, H_2_ was not produced at a rate that would allow the methanogen to divide before the entire reactor was turned over in 9.9 h. The total amount of biomass in the reactor did not change with increased loading, and while the planktonic populations began to washout, biomass was balanced by growth in the biofilm (**Figure 6B**). Under these conditions, biomass distribution in the biofilm shifted toward a greater percentage of *D. vulgaris* that increased by nearly 25% from 16.5 to 20.0 mg (**Figure 6B**), and this resembled pre-steady-state (i.e., when lactate was not entirely consumed) population structure most likely as a result of the increased loading rate for lactate (∼3 mM h^-1^; **Table 1**). Despite the altered population ratio, the macrostructure of the biofilm remained similar to the steady-state structure (lactate loading rate ∼0.5 mM h^-1^) with a *D. vulgaris* matrix intermixed with *M. maripaludis*.

In spite of lactate-excess, H_2_ was still undetectable (limit of detection ∼0.0001 mM or 0.25 Pa) when the loading rate was increased. This is contrary to published results for excess lactate planktonic-only conditions where H_2_ was continually 5–10 Pa at steady-state (Walker et al., 2009). These results suggest that the presence of biofilm caused a more efficient consumption or transfer of the produced H_2_ gas between *D. vulgaris* and *M. maripaludis*. Biomass yield per methane produced (mg protein/mM CH_4_) was significantly lower in lactate-excess conditions than in lactate-limited conditions (**Figure 6A**) and the same was true for biomass yield per lactate mass flux (mg protein/mM h^-1^ lactate). Essentially the same amount of biomass or carrying capacity was actively maintained under both conditions (i.e., lactate-excess versus lactate-limited) but the population distributions were different, and biofilm was able to increase metabolic flux without an increase in biomass. The carrying capacity, K, is defined as the maximum potential population size a given landscape is capable of supporting and is a common attribute used to describe population dynamics in ecology (Stilling, 2003; Berck et al., 2012). When additional lactate was available via an increased loading rate, the system was perturbed (washout of planktonic biomass) but the total biofilm biomass increased (**Figure 6B**). The total carrying capacity thus remained the same even though the population distribution and metabolic flux changed under lactate-excess conditions.

### BIOFILM REMOVAL

In a separate biofilm reactor at steady-state, the biofilm coupons were removed after 432 h (**Figure 4B**) to test the stability and population structure of the planktonic community in the absence of biofilm. Upon biofilm removal, the planktonic optical density increased within 24 h, but methane levels did not increase for 50 h (**Figure 4B**). After a 50-h static period, methane concentrations increased rapidly but declined back to original steady-state levels (15 h time period). Lactate and H_2_ were not detected, the OD increased, and similar levels of methane were produced as the system attempted to reach a new steady-state. The planktonic-phase only reactor population was 82% *D. vulgaris* but both *D. vulgaris* and *M. maripaludis* increased in absolute number based on cell counts and OD (**Table 1** and **Figure 4B**). Although 1.4 times greater biomass was maintained in the planktonic phase alone than the planktonic phase of the base case, the total reactor biomass was 3.4 times lower than the total biofilm plus planktonic biomass in the base case reactor (**Figure 6B**). The biomass yield was significantly lower than in the base case, and similar to total biomass yields in lactate-excess conditions (**Figure 6A**). Without a biofilm community, the carrying capacity of the system was significantly reduced under lactate-limiting conditions and the population distribution was less even.

To further investigate the role of biofilm in carrying capacity and stability, a mutant *D. vulgaris,* Δ*pilA*, was grown in coculture with wild-type *M. maripaludis*. The Δ*pilA D. vulgaris* lacks a presumptive type IV pilus and is deficient in biofilm formation (Figure S5). In batch coculture experiments, total methane production and growth were the same as wild-type coculture (data not shown). However, in continuous culture, the Δ*pilA* coculture did not form biofilm and the coculture grew as a planktonic-phase with similar levels of methane produced compared to wild-type coculture (0.09 mM at steady-state; **Figure 4C**). At 175 h, the biomass yield of Δ*pilA* coculture was similar to the planktonic-only wild-type coculture reactor (**Figure 6A**), and the total biomass was slightly higher than planktonic-only wild-type coculture (**Figure 6B**) with a similar distribution of *D. vulgaris* (**Table 1**). However, the mutant coculture did not stabilize and completely washed out of the reactor within 400 h (**Figure 4C**). In addition, in contrast to wild-type, H_2_ was detectable (50–120 Pa) over the whole 350 h of continuous culture until cells were too few to count in the planktonic phase (**Figure 4C**). This result further demonstrates the role of biofilm structure in facilitating and stabilizing syntrophic interactions. In the absence of biofilm (either physically removing the biofilm or biofilm deficient coculture), the community was not stable, H_2_ production and consumption were not balanced, and the carrying capacity declined for the methanogen.

In another δ-*Proteobacterium*, *Geobacter sulfurreducens*, PilA has been shown to be involved in extracellular electron transfer and biofilm formation (Richter et al., 2012), while in an aerobic δ-*Proteobacterium*, *Myxococcus xanthus*, PilA was shown to interact with biofilm EPS (Wei et al., 2012). Further work is needed to determine the role of type IV pili in facilitating interactions between SRB and hydrogenotrophic methanogens, but it seems likely that the pilus functions in attachment of cells to surfaces (biotic or abiotic) that directly or indirectly facilitates metabolic exchange.

## DISCUSSION

The primary objective of this work was to characterize the relationship between function and structure of a syntrophic biofilm community. Previous work suggests that specific structural patterns can be expected in interacting communities (Nielsen et al., 2000; Gu et al., 2013; Momeni et al., 2013), and that these patterns are dependent upon the nature of the interaction. We observed a structured syntrophic biofilm with complex ridges and channels, where both partners were highly intermixed. Similar biofilm structures have been observed in mixed communities and are also presumed to be a direct result of interaction type (Nielsen et al., 2000; Molin and Tolker-Nielsen, 2003), so it is reasonable to expect that structure affects community function and vice versa. Several results reported here indicate that syntrophic lactate oxidation and transfer of the H_2_ intermediate dictated the biofilm structure. Biofilm structures were never observed to exceed 50 μm in at least one dimension, and the BAM for this community predicted that biofilm thicker than 50 μm would experience lactate diffusion limitation at the substratum. These results suggest that lactate diffusion governed *D. vulgaris* biofilm structure. This type of structure would also have the same positive effect on H_2_ diffusing away from the SRB, where a buildup of the inhibitory by-product would prevent further lactate oxidation. Monoculture biofilms of *D. vulgaris* grown with sulfate and lactate similarly form only thin biofilms, yet they do not form tall structures in the way that syntrophic *D. vulgaris* does. These results suggest that syntrophic interactions drive the observed structural features.

When we consider the structure–function relationship from the point of the methanogen, the results suggest that the biofilm structure was driven by H_2_. In monoculture with H_2_, *M. maripaludis* did not form biofilm, however, when grown syntrophically the methanogen did join the H_2_-producing SRB biofilm. While it is possible that *D. vulgaris* biofilm simply provided a more suitable surface for attachment of *M. maripaludis*, it is also very likely that H_2_ drove the interaction specifically. We have recently shown chemotaxis toward H_2_ gas (hydrogenotaxis) in *M. maripaludis* (Brileya et al., 2013) and the response is especially strong under H_2_-limited conditions. H_2_ produced by SRB in the biofilm could diffuse to the aqueous and gas phases of the reactor, making it possible for planktonic *M. maripaludis* to scavenge the energy source without joining the biofilm. In spite of this option, more methanogen biomass was observed in the biofilm than the planktonic phase, whenever biofilm was present in the system. This suggests that some benefit is gained by interacting directly or closely with the SRB in the biofilm. These observations are supported by the lack of H_2_ detected during cultivation of wild-type populations as coculture biofilm. The results of the mutant coculture experiment further support a benefit from close interaction, since the mutant SRB lacked a pilus that presumably helped interactions directly through attachment or motility in the biofilm. The lack of direct interaction in biofilm resulted in detectable H_2_, and therefore inefficient transfer of the intermediate. It was recently shown that motility is an important determinant for structuring mixed biofilms when a motile *Bacillus* could infiltrate a *Staphylococcus* biofilm (Houry et al., 2012).

Analysis of cell-associated carbohydrates showed that *M. maripaludis* likely reallocates carbon in the syntrophic biofilm, given that the coculture biofilm had 10-fold less cell-associated carbohydrate than a monoculture *M. maripaludis* pellicle. One possible explanation is that a thick extracellular matrix would increase H_2_ mass transfer resistance, so the methanogen produced less carbohydrate to facilitate in H_2_ diffusion through the biofilm. Another possible explanation for altered carbon allocation is that *M. maripaludis* produced less EPS when grown as syntrophic biofilm to facilitate repositioning within the biofilm. *M. maripaludis* has only been shown to sense and swim toward H_2_ gas in liquid (Brileya et al., 2013), but it has not been shown to swarm, so it remains unclear whether individuals could move through a dense EPS matrix toward a higher concentration of H_2_.

Pure mutualism refers to the fact that the relationship is obligate and the growth rates of both populations are limited only by the concentrations of critical substrates produced by the partner (Meyer et al., 1975). The case of a SRB that oxidizes organic carbon to H_2_ and a methanogen that consumes the H_2_ is a variation that can be termed mutualism via product inhibition (Dean, 1985). Historically, microbial interactions have been studied in terms of competition for substrate and the competition coefficients are typically a ratio of the yield coefficients (Dean, 1985). Thus, stable or even unstable equilibria do not exist in terms of one population ‘winning’ over the other. However, these equations are based upon chemostats with only bulk-phase populations and not biofilms with inherent variability. Positive interactions could stabilize many more microbial interactions than previously thought (Shindala et al., 1965; Megee et al., 1972), particularly for biofilms. Our results support that community stability is a result of syntrophic interaction in biofilm. When lactate-loading rate was increased, causing washout conditions in the aqueous phase, biomass in the biofilm increased while planktonic biomass decreased. The washout situation highlights a complication of mutualistic interaction under flow conditions in which a population with a slower specific growth rate produces the limiting substrate of another population. Biomass retention in biofilm and close interaction represent logical ecological solutions to this problem. Community members are able to stay in a desirable location, rather than be washed away to potentially unfavorable environments. The mutant coculture could not form biofilm, and possibly close interactions, and therefore was unable to form a stable syntrophy with tight coupling between H_2_ production and consumption.

Macroecologists and microbial ecologists alike have modeled mutualism to gain insight into inter-population dynamics, and results predict stability of cooperative populations under only specific density-dependent conditions (Boucher, 1988). Experiments with mutualistic microorganisms have revealed many adaptations to syntrophic relationships, including alternative electron-transport pathways, differences in gene expression patterns in the presence of a syntrophic partner, and rapid evolution resulting in optimized biomass production (Shimoyama et al., 2009; Walker et al., 2009, 2012; Hillesland and Stahl, 2010; Plugge et al., 2011; Bernstein et al., 2012; Lawrence et al., 2012; Sieber et al., 2012). In this syntrophic system, while we observed that biofilm growth mode promoted the greatest biomass retention and allowed the system to reach increased carrying capacity, we also observed that this biomass was metabolically functional, in spite of lactate limitation. CTC staining and FISH indicated that the whole biofilm biomass had respiratory potential. When additional lactate was added via an increased loading rate, the biofilm community responded within 1 h by increasing electron flux from lactate to methane. It is quite interesting to consider this result in the context of a low-nutrient environment, where it seems likely that a natural biofilm community could remain poised for episodic nutrient availability. Our results indicate that in a mixed community, syntrophs are able to rapidly cycle electrons or carbon.

In this model syntrophic system, structured biofilm promoted maximum carrying capacity, contributed to cooperative resource sharing (i.e., improved H_2_ transfer) and provided greater community stability when compared to planktonic-only populations. Although both biofilms and syntrophic communities are inherently variable and heterogeneous, these culture conditions are environmentally relevant. Mixed culture biofilm reactors can be used to experimentally explore ecological and evolutionary phenomena in a more constrained setting. It remains to be seen what genetic and metabolic controls are responsible for the observed responses in this system, and future work is planned to understand how specific biofilm structures and interactions can impact meso- and macro-scale processes including greenhouse gas production, biogeochemical cycling, and waste conversion.

## AUTHOR CONTRIBUTIONS

Kristen A. Brileya designed and performed experiments, analyzed and interpreted data, drafted and revised the manuscript. Laura B. Camilleri performed experiments, analyzed and interpreted data, and revised the manuscript. Grant M. Zane designed and performed experiments to create the mutant Δ*pilA D. vulgaris,* and revised the manuscript. Judy D. Wall designed experiments to create the mutant Δ*pilA D. vulgaris*, interpreted the data for this manuscript, and made critical revisions to the manuscript. Matthew W. Fields designed experiments, interpreted data, drafted and revised the manuscript.

## Conflict of Interest Statement

The authors declare that the research was conducted in the absence of any commercial or financial relationships that could be construed as a potential conflict of interest.
