# Supplementary material for: Biofilm growth mode promotes maximum carrying capacity and community stability during product inhibition syntrophy
Source: Front Microbiol. 2014 Dec 15;5:693. doi: 10.3389/fmicb.2014.00693 (PMC4266047; doi:10.3389/fmicb.2014.00693)
Supplement: Supplementary file 1 [file Data_Sheet_1.PDF]

Supplemental figures and legends

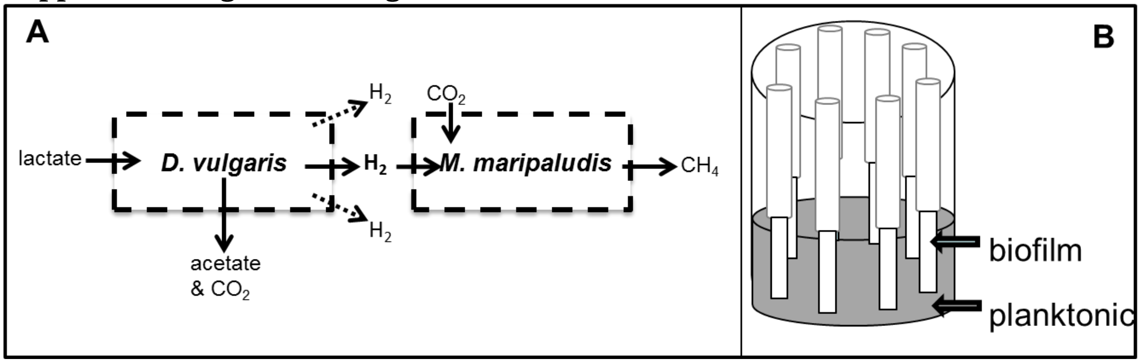

Supplemental Figure 1. Schematic of experimental system (**A**) showing the nature of this syntrophic interaction where *D. vulgaris* oxidizes lactate to produce H_2 which is then used by *M. maripaludis* to reduce CO_2 to CH_4. Consumption of H_2 by the methanogen prevents product inhibition where a large pool of H_2 would inhibit lactate oxidation by the SRB. (**B**) showing how glass biofilm coupons are suspended in the planktonic phase culture medium.

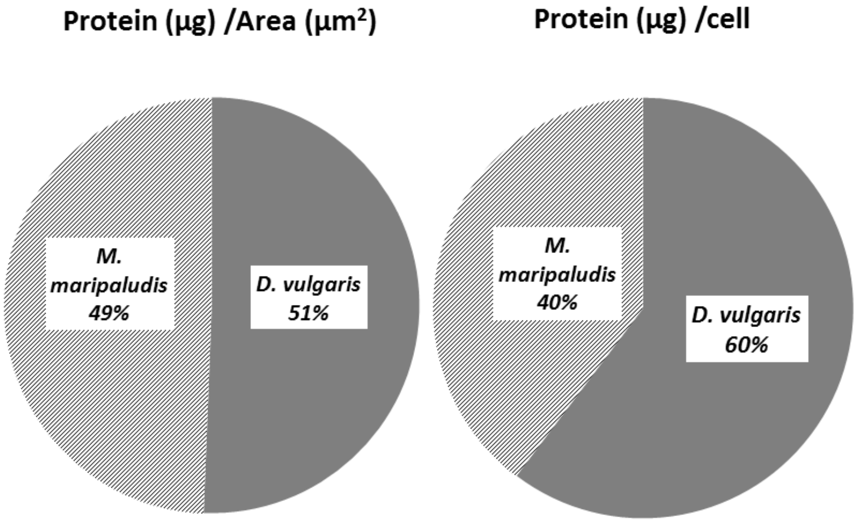

	Protein (pg)/cell	Protein/Area (pg/μm^2)
D. vulgaris	949 ± 563	402 ± 255
M. maripaludis	625 ± 281	392 ± 134

Supplemental Figure 2. Average protein per cell and per thresholded area for monocultures of *D. vulgaris* and *M. maripaludis* and 95% confidence interval. These ratios were used to adjust coculture planktonic protein amounts per organism based on cell counts.

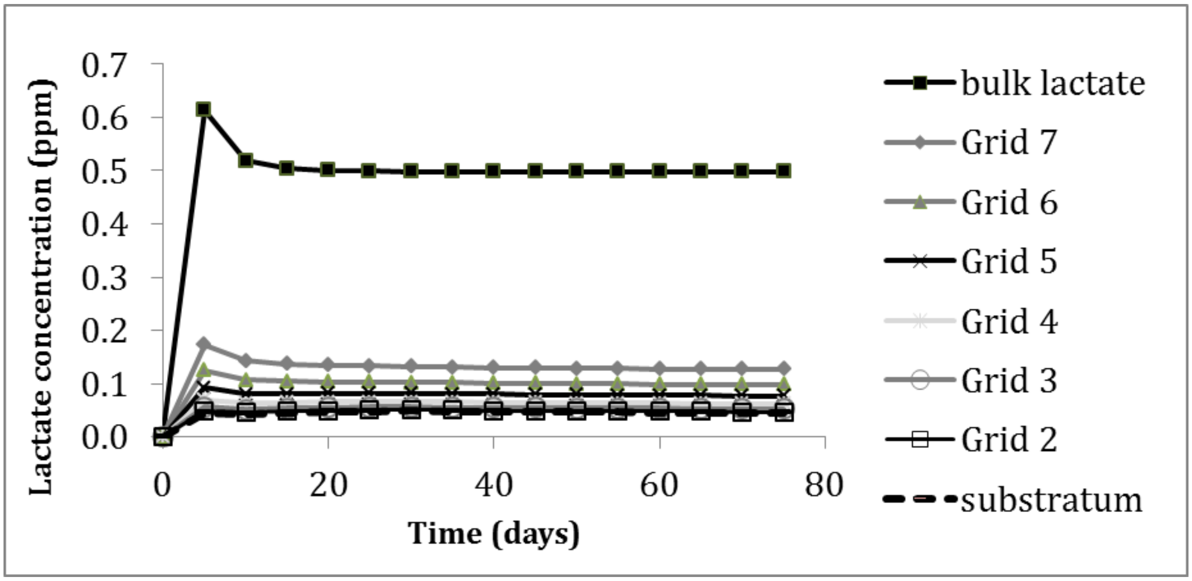

Supplemental Figure 3. Lactate concentration (ppm) predicted by the Biofilm Accumulation Model in the aqueous (bulk) phase and at multiple depths in the biofilm, including the bottom (substratum).

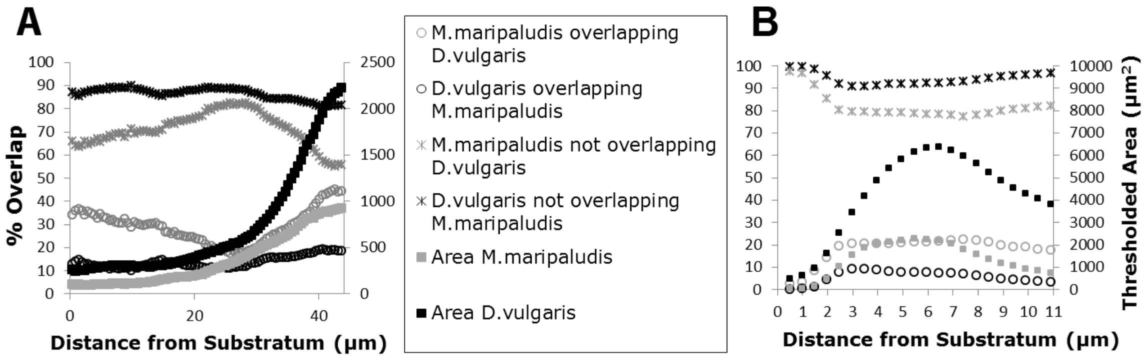

Supplemental Figure 4. Percent of *D. vulgaris* and *M. maripaludis* cells overlapping (O) and not overlapping (*) (primary axis) and total thresholded area of each (□) (secondary axis) in a vertical section of (**A**) intermediate (48 hr) (**B**) steady-state coculture biofilm.

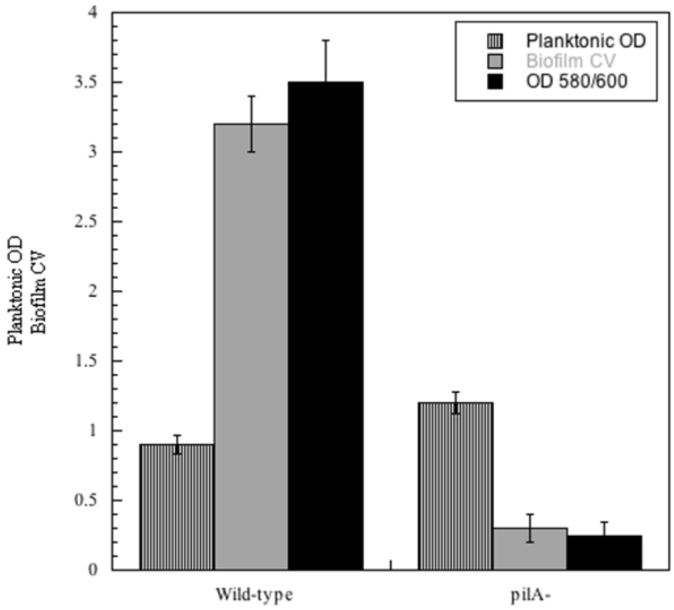

Supplemental Figure 5. Biofilm produced by wild-type *D. vulgaris* versus mutant Δ*pilA* *D. vulgaris* quantified by Crystal Violet (CV) staining of whole biofilm coupons as previously described (Clark *et al*., 2007). Values represent the ratio of absorbance of the CV destaining solution OD_{580} versus the planktonic phase OD_{600}.

Area	.0591 m^2
Volume	3.5 x 10^{-4} m^3
Volumetric Flow Rate	.000126 $m^3 day^{-1}$
μ_{max}	.72 day^{-1}
$S_{lactate}$	2702.24 g/m^3
S_{CO2}	5000g/m^3
De lactate	1.83 x 10^{-5} m^2/day
De CO_2	1.13 x 10^{-4} m^2/day
De H_2	2.65 x $10^{-4}m^2$/day
De CH_4	8.76 x 10^{-5} m^2/day
Cell Growth	First Order
Substrate consumption	First Order
Substrate production	Zero Order

Supplemental Table 1. Input parameters for Biofilm Accumulation Model.
